# Optogenetics to help exploring the cerebral blood flow regulation

**DOI:** 10.3389/fphar.2014.00107

**Published:** 2014-05-13

**Authors:** Piotr Bregestovski, Yuri Zilberter

**Affiliations:** Institut de Neurosciences des Systèmes, INSERM UMR1106Marseille, France

**Keywords:** neuro-vascular coupling, optogenetic approach, neuronal networks, BOLD, interneurons

Mammalian brain contains a rich vascular network. This network's functional activity, i.e., blood flow, is tightly controlled by neural networks, although the mechanisms of this control are not yet clear. Understanding the link between neuronal activity and cerebral blood flow (CBF) is essential for both theoretical and applied aspects of brain vascular dysfunctions in neurodegenerative diseases (de la Torre, 2012). Optogenetic techniques provide unique opportunities for monitoring and modulating functions of specific neurons and neuronal networks. An excellent example is the recent study of Urban et al. (2012) on regulation of CBF in the mouse brain.

Up till now, three main hypothesis have been put forward to explain neuro-vascular coupling: *metabolic, hemo-neuronal*, and *neurogenic*. The *metabolic* hypothesis originates from the seminal study of Roy and Sherrington proposing that the regional CBF is regulated by local metabolites (Roy and Sherrington, 1890). Since that time, a number of studies suggested a causal link between neuronal energy demands and regulation of the local CBF (Hamel, 2006). The *metabolic* hypothesis suggests that neurovascular coupling is mediated by chemical signals released from activated perivascular nerve endings and astrocytes. These signals alter vascular tone in order to locally adjust blood supply according to the demands of neuronal activity. This insures the CBF coupling with regional glucose utilization, which in turn is directly related to neuro-glial activity (Magistretti, 2006).

The *hemo-neuronal* hypothesis proposes that hemodynamics play a role in information processing via modulation of neural activity by blood flow. It suggests that functional hyperemia, the “overflow” of blood to a brain region during neural activity, provides a spatially and temporally correlated source of regulation, modulating the excitability of the local circuit (Moore and Cao, 2008).

The *neurogenic* hypothesis suggests that neuronal synaptic signaling (rather than metabolic needs) initiates a feed-forward link to cerebrovascular reactions (Estrada and Defelipe, 1998). This hypothesis is supported by the observation that in the brain slices from frontopariental cortex, activation of a single interneuron is sufficient to induce the vasomotor response in local microvessel network (Cauli et al., 2004). This suggests the ability of specific subsets of cortical GABA interneurons to translate neuronal signals into vascular responses. Moreover, it has been proposed that local activation of neurons and astrocytes leads to a change in tone of smooth muscles in surrounding arterioles thus precisely modulating the blood flow in a particular capillary bed (Kleinfeld et al., 2011).

To test the last hypothesis, Urban et al. (2012) used the optogenetic tools for activating specific local neuronal networks and monitoring whether this causes changes in a diameter of blood vessels in the mouse brain slices. Their strategy was based on the following main approaches. First, they used a mutant of ChR2-EYFP that has faster kinetics, which allowed them to study the effects of optogenetic stimulation over a broad range of frequencies (up to 200 Hz). Second, they used a Cre-dependant virus containing ChETA-EYFP expression cassette under the control of strong EF1α promoter that ensured a high level of expression. Finally, to restrict expression of ChR2-EYFP to specific cells the authors used transgenic mouse expressing Cre recombinase in neurons containing parvalbumin (PV).

For monitoring the light-induced currents, the authors performed whole-cell patch-clamp recordings and ascertained that the fast spiking-PV interneurons reliably fired action potentials in response to the patterned blue light stimulation. Brain slice preparation allowed the direct visualization of microvessel movements and contractions. For analysis of vascular reactivity, the blood vessels of a >50 μm length and 8–30 μm luminal diameter were selected. Using infrared video microscopy, the authors demonstrated that the optogenetic stimulation of PV-positive neurons lead to a decrease in the diameter of arterioles, indicating that photo-stimulation of PV- containing neurons efficiently induces a contraction of penetrating arterioles. One important finding was that a subclass of PV-expressing pyramidal neurons might also be a part of the neuronal system that controls local blood flow by inducing vasoconstriction. The authors conclude that signaling from either fast spiking-PV interneurons or PV-positive pyramids could be involved in vasoconstriction of penetrating arterioles.

The more we know about neurovascular coupling, the more we understand that this extremely complex mechanism cannot be explained within the frames of any unidirectional hypothesis. Most likely, this process reflects the combination of feedback (metabolic) and feed-forward (signaling) pathways. Moreover, both pathway-mediated mechanisms closely interact in regulation of blood flow. From the perspective of neuronal function, the hemodynamic signals may reflect both neuronal inhibition and excitation, thus, for a given brain area, the polarity of blood oxygen level-dependent (BOLD) fMRI signals may critically depend on the type of synaptic input and therefore on the balance between vasodilator and vasoconstrictor molecules released by excitatory and inhibitory neurons and astrocytes. Up to now, there is no straightforward interpretation of a positive or negative BOLD signal in terms of the underlying neuronal activity and hence on the type of neurons that are activated. Taking into account the complexity of the problem, it is difficult to overestimate the importance of optogenetic approach since this technique provides direct information on the link between activity of certain subclass of neurons and correlated response of the vascular system. Combination of functional Magnetic Resonance Imaging (fMRI) with optogenetics has been successfully used for mapping neuronal connectivity in the hippocampal formation (Abe et al., 2012) and in other domains (Desai et al., 2011). In the future, optogenetics in combination with electrophysiological (e.g., EEG, MEG) and imaging tehniques (e.g., fMRI) promises to provide a means to track changes in the intact brain activity elicited by defined neuronal populations and determine the contributions of different cell types in the network to the BOLD signal.

## Conflict of interest statement

The authors declare that the research was conducted in the absence of any commercial or financial relationships that could be construed as a potential conflict of interest.
